# Morphological, physiological, and root metabolomic responses of *Hemerocallis minor* Mill. to drought stress

**DOI:** 10.3389/fpls.2026.1855949

**Published:** 2026-07-16

**Authors:** Zhen Liu, Zichan Li, Can Wang, Yikang Tian, Qiqi Han, Xiaoming Wan, Zhongkuan Liu, Zhenyu Liu, Guixia Liu

**Affiliations:** 1 School of Life Sciences, Hebei University, Baoding, China; 2 Key Laboratory of Regional Energy and Environmental Systems Optimization, Ministry of Education, North China Electric Power University, Beijing, China; 3 Institute of Agro-resources and Environment, Hebei Academy of Agriculture and Forestry Sciences, Shijiazhuang, China

**Keywords:** drought tolerance, isoflavonoids, morphophysiological adaptation, root metabolic profiling, water deficit

## Abstract

**Introduction:**

Drought stress is a major environmental factor limiting plant growth and ecosystem stability in arid and semi-arid regions. *Hemerocallis minor* Mill., a perennial herb with strong drought tolerance, has potential applications in ecological restoration and marginal land utilization. However, the coordinated relationships among morphological, physiological, and metabolic responses, as well as the response characteristics under different intensities of drought stress, remain poorly understood.

**Methods:**

In this study, a 12-week gradient drought stress experiment was conducted. An integrated multi-parameter approach was applied to systematically evaluate the responses of *H. minor* to varying drought intensities at the levels of growth morphology, physiological traits, and root metabolism.

**Results:**

Morphologically, drought stress induced changes such as root thickening, whereas severe and extreme drought significantly inhibited plant height, root growth, and adventitious root formation. Physiologically, plants under moderate drought maintained relatively stable leaf water content and some physiological traits, while severe drought caused pronounced membrane damage and oxidative stress responses. Metabolically, drought stress substantially remodeled root metabolic networks, with numerous differential metabolites detected across comparison groups, primarily involving amino acids, flavonoids, and coumarins. Among them, 23 differential metabolites were annotated to the isoflavonoid biosynthesis pathway. Correlation analysis indicated significant associations between root morphological traits, physiological indicators, and key metabolites: proline, malondialdehyde (MDA), and peroxidase (POD) activity were negatively correlated with certain growth parameters, whereas some isoflavone glycosides and isoflavonoid metabolites were positively correlated.

**Discussion:**

This study provides a comprehensive “morphology-physiology -metabolism” perspective on the responses of *H. minor* to drought stress. Results indicate that under mild to moderate drought, plants maintain relatively stable growth and physiological status, whereas severe drought induces pronounced stress responses and metabolic adjustments. These response patterns likely contribute to the adaptation of *H. minor* to drought environments. The findings offer references for the selection and application of drought-tolerant plants in ecological restoration and marginal land utilization, while further validation of the underlying response mechanisms and ecological significance is warranted through anatomical, molecular, targeted metabolomic, and field studies.

## 1 Introduction

Water is an essential resource for plant survival, and plant organs must maintain sufficient water content to ensure normal physiological activities and growth (Podzikowski et al., 2025). However, global climate change has significantly altered precipitation patterns, leading to the increasing occurrence of extreme drought events and the aggravation of water scarcity worldwide (Zhang et al., 2025). According to the Food and Agriculture Organization of the United Nations (FAO), drought can reduce global crop yields by 10%-30%, posing severe threats to food security, agricultural sustainability, and ecosystem stability (Freedman et al., 2025). Drought stress generally refers to a series of growth, physiological, and metabolic responses induced by insufficient soil moisture, which can inhibit photosynthesis, disrupt cellular water balance, and promote excessive accumulation of reactive oxygen species (ROS), thereby adversely affecting plant growth and development (Ali et al., 2025). It is important to distinguish three related but hierarchically distinct concepts under drought conditions: drought response emphasizes short-term physiological and metabolic changes, drought tolerance focuses on the ability to maintain growth and survival, and drought adaptation generally refers to long-term evolutionary or stable trait adjustments to environmental stress. These processes are interconnected but should not be treated as equivalent (Haghpanah et al., 2024). To adapt to water-deficient environments, plants have evolved multi-level drought response and adaptation strategies, including root morphological plasticity, osmotic adjustment, antioxidant defense, and metabolic reprogramming (Zhang et al., 2024; Shen et al., 2025). Among these mechanisms, roots are regarded as key organs for sensing and responding to soil water fluctuations, and changes in root structure and function play crucial roles in plant drought adaptation. Previous studies have demonstrated that under moderate drought conditions, some plants can enhance water acquisition capacity by promoting root elongation, root thickening, and increasing the root-to-shoot ratio, whereas severe drought stress often suppresses root growth and decreases root activity (Ranjan et al., 2022). In addition, plants may alleviate oxidative damage through the accumulation of osmotic adjustment substances such as proline and soluble sugars, as well as by regulating the activities of antioxidant enzymes including superoxide dismutase (SOD) and peroxidase (POD) (Wang et al., 2024a; Song et al., 2025). Nevertheless, it should be noted that changes in proline content, malondialdehyde (MDA) accumulation, and antioxidant enzyme activities mainly reflect plant stress response characteristics and do not necessarily indicate enhanced drought tolerance. Whether these responses truly contribute to drought adaptation should be comprehensively evaluated in combination with plant growth maintenance, survival status, and recovery capacity.

In recent years, with the rapid development of metabolomics technologies, increasing evidence has shown that plants undergo substantial metabolic reprogramming under drought conditions, involving the regulation of amino acids, flavonoids, phenolic acids, terpenoids, and other metabolic pathways associated with drought adaptation (Wang et al., 2024b). For example, Yadav et al. (2019) reported that wheat (*Triticum aestivum*) accumulated osmotic adjustment-related metabolites such as proline and histidine under drought stress. Similarly, Khan et al. (2019) found that long-term drought stress significantly altered the metabolomic profile of chickpea (*Cicer arietinum*). Morcol et al. (2020) further demonstrated that secondary metabolites including phenylpropanoids, flavonoids, and fatty acids changed significantly under drought stress and may participate in ROS scavenging, osmotic buffering, and stress adaptation. In addition to metabolic regulation, some plant species exhibit morphological changes such as root thickening or root succulence under drought stress. Current studies generally suggest that these structural modifications may be associated with enhanced water storage capacity and carbohydrate accumulation; however, their specific ecophysiological functions and underlying formation mechanisms remain uncertain, and no consensus has yet been reached (Kou et al., 2022). Some researchers have proposed that succulent roots may contribute to drought responses by improving tissue water status and buffering short-term fluctuations in water availability, although whether such traits represent stable adaptive structures requires further verification. Furthermore, these morphological changes may occur concurrently with metabolic adjustments and antioxidant responses, but the mechanistic links among these processes remain poorly understood (Ranjan et al., 2022). However, the specific physiological and ecological roles of succulent roots in drought adaptation and their relationships with metabolic regulation remain insufficiently understood, particularly in *Hemerocallis* species.

*Hemerocallis minor* Mill., a perennial herb belonging to the genus *Hemerocallis* in the family Asphodelaceae, is mainly distributed in arid and semi-arid regions of northern China and possesses strong tolerance to drought, poor soils, and saline-alkaline conditions. The species develops a well-developed root system with obvious succulent characteristics, suggesting considerable potential for ecological restoration, marginal land utilization, and industrial ecological vegetation construction. In addition, its flower buds and young leaves are rich in proteins, amino acids, and mineral elements, making it valuable for food processing and ornamental horticulture (Bai, 2023). At present, studies on drought responses in *Hemerocallis* species mainly focus on leaf physiological characteristics and antioxidant enzyme activities. Previous research has shown that *H. minor* can maintain a relatively stable root-to-shoot ratio under drought stress, accompanied by lower leaf water loss rates, increased POD activity, and MDA accumulation. Compared with *Hemerocallis fulva* and *Hemerocallis citrina*, *H. minor* exhibits stronger environmental adaptability in terms of root growth and antioxidant system regulation (Wang, 2018). Nevertheless, existing studies mainly emphasize individual physiological indicators, while systematic investigations on the relationships among root morphological plasticity, root metabolic reprogramming, and multi-level drought responses remain limited.

Notably, preliminary field observations suggested that the roots of *H. minor* may exhibit thickening and succulence under drought conditions. However, direct evidence regarding the formation of these traits under different drought intensities and their ecological functions remains limited. Previous studies have proposed that succulent roots may be associated with temporary water storage, carbon metabolic adjustment, and stress-buffering processes; however, these proposed functions are largely based on hypotheses or indirect observations and have not been systematically validated (He et al., 2013; Leufen et al., 2016). Therefore, a systematic investigation of the drought response characteristics of *H. minor* from a multi-level perspective encompassing root morphological changes, physiological responses, and metabolic regulation is important for improving our understanding of its drought responses and their potential adaptive significance.

Based on these considerations, *H. minor* was subjected to a gradient drought stress experiment. Growth morphology, physiological traits, and root metabolites were comprehensively analyzed to characterize its responses to different drought intensities, with particular attention given to root succulence and its associations with physiological and metabolic responses. The study aimed to elucidate the multi-level drought response patterns of *H. minor* and explore their potential adaptive significance. The findings may provide useful references for drought-tolerant plant selection, ecological restoration, and vegetation establishment on marginal or drought-prone lands.

## 2 Materials and methods

### 2.1 Experimental materials

The experiment was conducted in the intelligent greenhouse of the Innovation Research Base of Chengde Academy of Agriculture and Forestry Sciences, Longhua County, Chengde City, Hebei Province, China. During the experimental period, the greenhouse temperature was maintained at 22-28 °Cduring the daytime and 16-20 °Cat night, with relative humidity maintained at 55%-70%. Natural light was used as the primary light source, with an approximate photoperiod of 14 h light/10 h dark. The greenhouse was regularly ventilated to maintain air circulation. To minimize environmental variation, planting bags from different treatments were randomly arranged and periodically rotated during the experiment.

The experimental material was the wild type of *H. minor*, a perennial herb belonging to the genus *Hemerocallis* of the family Asphodelaceae. Uniform and healthy two-year-old plants without visible pests or diseases were selected from the wild flower germplasm nursery of the Chengde Academy of Agriculture and Forestry Sciences.

### 2.2 Experimental design

Thirty actively growing *H. minor* plants were transplanted into planting bags (40 cm in length X 50 cm in height), with one plant per bag. The growth substrate consisted of conventional greenhouse cultivation soil, which was classified as loam soil with a pH of 7.2 and an organic matter content of 18.6 g·kg^-1^. After transplantation, all plants were watered normally for two weeks to allow acclimation before drought stress treatments were initiated (Geng et al., 2021).

The maximum water-holding capacity of the soil was determined to be 34%. Soil maximum water-holding capacity was measured using the indoor saturated gravimetric method. Air-dried soil was fully saturated with water and allowed to drain naturally for 24 h before weighing. The soil was then oven-dried at 105 °C to constant weight, and the maximum water-holding capacity was calculated based on the saturated water content and dry soil mass. To systematically investigate the growth, root morphology, and physiological-metabolic responses of *H. minor* under different drought intensities, and to cover the full range from near-sufficient water to extreme drought while ensuring comparability between treatments and manageable experimental conditions, five soil moisture treatments were established as follows:

1. Control group (CK): 75%‐80% of the maximum water holding capacity.
2. Mild drought stress (W1): 55%‐60% of the maximum water holding capacity.
3. Moderate drought stress (W2): 45%-50% of the maximum water holding capacity.
4. Severe drought stress (W3): 25%-30% of the maximum water holding capacity.
5. Extreme drought stress (W4): 15%-20% of the maximum water holding capacity.

**Figure 1:**
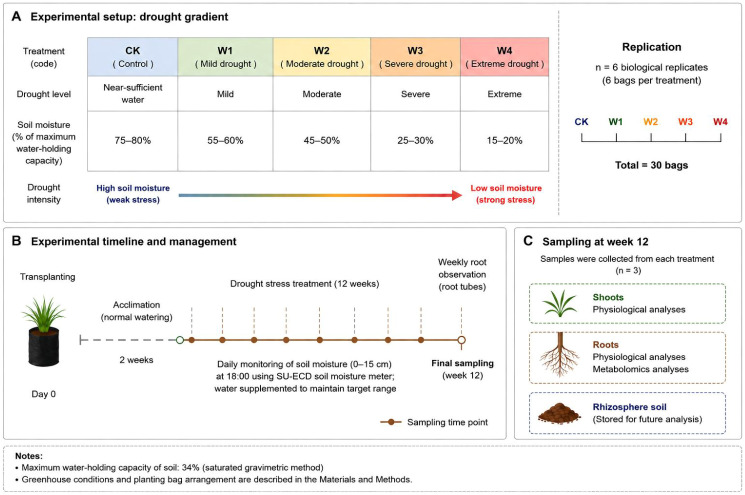
Overview of the experimental design for drought stress treatments in Hemerocallis minor. **(A)** Experimental setup showing the drought gradient treatments (CK, W1, W2, W3, and W4), soil moisture levels, drought intensity, and biological replication. **(B)** Experimental timeline showing plant acclimation, 12-week drought treatment, soil moisture monitoring, and final sampling. **(C)** Sampling scheme at week 12 showing the collection of shoots, roots, and rhizosphere soil for physiological and metabolomic analyses.

Each treatment consisted of six replicates, with a total of 30 planting bags. The target soil moisture range for each treatment was calculated based on the maximum water-holding capacity. During the experiment, soil moisture content within the 0–15 cm soil layer of each planting bag was measured daily at 18:00 using an SU-ECD intelligent soil moisture meter. Three measurements were randomly taken from each bag and averaged. Water was supplemented according to the measured values to maintain the target soil moisture range for each treatment as consistently as possible. The drought stress treatment period lasted for 12 weeks. During the experiment, root growth dynamics were monitored weekly using minirhizotron tubes, and the growth status and developmental trends of roots under different treatments were recorded to provide reference information for analyzing root response characteristics under drought stress. Because the quantitative analysis of root morphological parameters was based on root scanning measurements performed after harvest at the end of the experiment, the minirhizotron observations were used only as supplementary qualitative observations and were not included in the statistical analyses. Samples were collected after 12 weeks of drought treatment, when obvious root succulence had begun to appear in *H. minor*, indicating that the plants had developed adaptive responses to drought stress. During sampling, shoots, roots, and rhizosphere soils were separately collected into sterile bags for subsequent physiological and metabolomic analyses (Zhao et al., 2026). The detailed drought experimental design is shown in **Figure 1**.

### 2.3 Experimental methods

#### 2.3.1 Determination of growth and morphological traits

Growth and morphological traits of *H. minor* were measured at the 4th, 8th, and 12th weeks after drought treatment. Each treatment contained six biological replicates in total. Among them, three plants were selected for continuous plant height monitoring, and the remaining three were used for other destructive sampling or reserve purposes. Before the drought treatments began, three plants with relatively uniform growth were selected from each treatment as fixed observation subjects, and their plant height was continuously monitored throughout the experiment to minimize the influence of individual variation on dynamic change analysis. Plant height was measured using a measuring tape from the base of the plant to the tip of the tallest leaf, and growth rate was calculated based on changes in plant height at different sampling times (Li et al., 2023). The plant height data in **Table 1** are based on these three fixed observation plants (n = 3).

**Table 1:** Effects of drought stress treatment on plant height of *Hemerocallis minor*.

Treatment	Plant height at week 4 (cm)	Plant height at week 8 (cm)	Plant height at week 12 (cm)
CK	39.93 ± 3.04a	59.93 ± 3.55a	65.87 ± 3.29a
W1	44.83 ± 0.77a	60.20 ± 0.44a	68.47 ± 1.32a
W2	40.00 ± 0.71a	57.47 ± 2.42a	62.10 ± 2.97a
W3	28.80 ± 1.93b	39.23 ± 2.94b	41.93 ± 2.99b
W4	14.93 ± 1.13c	17.97 ± 1.30c	19.70 ± 1.51c

Values are presented as mean ± SD (n = 3). Different lowercase letters indicate significant differences among treatments at the same time point, determined by one-way ANOVA followed by Duncan’s multiple range test (*P* < 0.05).

Root morphological traits were analyzed using root tube observation combined with the RootSnap root analysis system. During the experiment, root growth dynamics were monitored weekly through *in situ* root tube observation. After 12 weeks of drought treatment, destructive sampling was conducted. Root systems were carefully removed from the planting bags and gently rinsed with distilled water to remove adhering soil while minimizing damage to fine roots. The cleaned roots were then spread flat on a white background board for image acquisition. Root morphological parameters, including total root volume (TRV), root surface area (RSA), average root diameter (ARD), total root length (TRL), and total root number (TRN), were analyzed using RootSnap software. Succulent roots were identified mainly based on morphological characteristics such as obvious root thickening, lighter root color, and tissue swelling (Zhou and Zeng, 1995). As this study focused on overall root morphological changes under different drought gradients, quantitative measurements of the number, proportion, or anatomical features of succulent roots were not performed. Therefore, related descriptions are based on morphological observations and do not involve functional interpretation.

#### 2.3.2 Determination of physiological parameters

Collected shoots and roots were rinsed with distilled water, gently blotted dry, rapidly frozen in liquid nitrogen, and stored at −80 °C prior to physiological analysis. Three biological replicates were established for each physiological parameter, and each replicate was independently analyzed.

For each assay, approximately 0.5 g (fresh weight) of sample was homogenized with the corresponding extraction buffer. Extraction solutions and reaction systems for each parameter were prepared according to the instructions of commercial kits or published methods. The extraction volume was generally 5-10 mL, adjusted as appropriate for specific assays.

Leaf and root relative water content (RWC) was determined using the fresh weight-saturated weight-dry weight method (Qiu et al., 2026). Soluble protein (SP) content was measured using the Coomassie Brilliant Blue G-250 method (kit purchased from Nanjing Jiancheng Bioengineering Institute) with bovine serum albumin as the standard; absorbance was read at 595 nm. For SP measurement, approximately 0.2 g of sample was homogenized in 3 mL phosphate buffer (pH 7.0), centrifuged at 12,000 rpm for 10 min, and the supernatant incubated at 37 °C for 10 min before measurement. Soluble sugar (SS) content was determined using the anthrone colorimetric method (Geng et al., 2024); 0.2 g of sample was extracted with 5 mL of 80 % ethanol in a water bath at 80 °C for 30 min, centrifuged at 10,000 rpm for 10 min, and the supernatant was used for the color reaction. Absorbance was measured at 620 nm.

Chlorophyll content was measured by extraction with an ethanol-acetone mixture (1:1, v/v). Approximately 0.1 g of leaf tissue was immersed in 10 mL of extraction solution in the dark for 24 h, and absorbance was read at 645 nm and 663 nm (Qi et al., 2026). Malondialdehyde (MDA) content was determined using the thiobarbituric acid (TBA) method (Wen et al., 2026). Approximately 0.2 g of sample was homogenized in 5 mL of 5 % trichloroacetic acid (TCA), centrifuged at 10,000 rpm for 10 min, and the supernatant was reacted with 0.67 % TBA solution in a 95 °C water bath for 30 min.

Superoxide anion (O_2_-) content was measured using the hydroxylamine oxidation method (Qi et al., 2025). Samples were extracted in phosphate buffer and incubated at 25 °C for 20 min; absorbance was measured at 550 nm. Relative electrical conductivity (REC) was determined using the immersion method (Wang et al., 2021). Leaf samples (0.2 g) were placed in 10 mL deionized water and incubated at 25 °C for 24 h to measure initial conductivity, followed by boiling for 30 min, cooling, and measurement of final conductivity.

Superoxide dismutase (SOD) activity was measured using the nitro blue tetrazolium (NBT) photoreduction method (kit from Suzhou Keming Biotechnology Co., Ltd.), and peroxidase (POD) activity was measured using the guaiacol method (kit from Suzhou Keming Biotechnology Co., Ltd.). Free proline (Pro) content was determined by the acid-ninhydrin colorimetric method (Li, 2000); approximately 0.5 g of sample was extracted with 5 mL of 3 % sulfosalicylic acid in a water bath at 100 °C for 30 min, centrifuged at 12,000 rpm for 10 min, and the supernatant reacted with ninhydrin, with absorbance measured at 520 nm.

All calculations of physiological parameters were conducted according to kit instructions or relevant literature formulas, and all results were expressed on a fresh weight (FW) basis.

#### 2.3.3 Determination of root metabolites

Root metabolomics analysis was conducted using a non-targeted metabolomics approach. Three biological replicates were selected for each treatment. Fresh root samples were immediately frozen in liquid nitrogen, ground into powder (~0.5 g fresh weight) under liquid nitrogen, transferred into 10 mL sterile centrifuge tubes, and stored at −80 °C for transportation to minimize metabolite degradation.

Metabolite extraction and LC-MS analysis were performed by Shanghai Meiji Biopharmaceutical Technology Co., Ltd. Samples were extracted using either a methanol:water:chloroform (2:1:1, v/v/v) system or a methanol/acetonitrile/water system according to platform-standard protocols. Internal standards (e.g., L-2-chlorophenylalanine) were added for data normalization. The extraction process was carried out by shaking at 4 °C for 30 min, followed by centrifugation at 12,000 rpm for 10 min, and the supernatant was used for analysis.

Chromatographic separation was performed on a UHPLC system (Thermo Vanquish or equivalent) equipped with a C18 reversed-phase column (2.1 mm × 100 mm, 1.7 μm). Mobile phase A was 0.1 % formic acid in water, and mobile phase B was acetonitrile. The gradient elution program was as follows: 0-2 min (5 % B), 2–12 min (5-95 % B), 12-14 min (95 % B), 14-16 min (5 % B for re-equilibration), with a flow rate of 0.3 mL·min^-1^ and an injection volume of 2-5 μL.

Mass spectrometry detection was conducted using a UHPLC-QTOF-MS or Orbitrap high-resolution MS platform, acquiring data in both positive and negative ion modes (ESI+/ESI−). The scan range was m/z 50-1200, with collision energy set at 20-40 eV for stepped energy fragmentation.

Data acquisition employed a data-dependent acquisition (DDA) mode to obtain MS/MS fragmentation information. To ensure system stability, one QC sample was injected after every ten experimental samples. Raw data were processed using XCMS, Progenesis QI, or Compound Discoverer software for peak extraction, alignment, and noise reduction, followed by normalization with internal standards and total ion current (TIC). Missing values were imputed using the half-minimum method, and batch effects were corrected using the QC-RLSC method or equivalent algorithms.

Metabolite annotation was based on HMDB, KEGG, and MassBank databases, combined with MS/MS fragment matching. Annotation confidence levels were assigned according to the Metabolomics Standards Initiative (MSI, Level 1-4), and only Level 2 or higher annotations were used for subsequent analysis. Differential metabolites were screened based on variable importance in projection (VIP > 1), Student’s t-test (*P* < 0.05), and fold change (FC ≥ 1.5 or FC ≤ 0.67), and the stability of the PLS-DA model was validated using 200 permutation tests (Lei et al., 2025).

### 2.4 Data processing

Raw data were organized and calculated using Microsoft Excel, and statistical analyses were performed using IBM SPSS Statistics 26.0 (Chicago, IL, USA). All results are presented as mean ± standard deviation (Mean ± SD). Prior to statistical analyses, data normality and homogeneity of variance were assessed using the Shapiro-Wilk test and Levene’s test, respectively. For endpoint variables, differences among treatments were evaluated using one-way analysis of variance (one-way ANOVA) when the assumptions of normality and homogeneity of variance were satisfied, followed by Duncan’s multiple range test for *post hoc* comparisons. Statistical significance was determined at *P* < 0.05.

Plant height was measured repeatedly on the same individuals at weeks 4, 8, and 12. Therefore, repeated-measures analysis of variance (repeated-measures ANOVA) was used to evaluate the effects of drought treatment, time, and their interaction on plant growth. In the repeated-measures analysis, the sphericity assumption was tested using Mauchly’s test. When the assumption of sphericity was violated, Greenhouse-Geisser corrections were applied to adjust the degrees of freedom and improve the robustness of statistical inference. Treatment effects, time effects, and treatment×time interactions were reported. Pearson correlation analysis was performed to evaluate relationships among growth morphology, physiological traits, and root metabolites, and the results were visualized using heatmaps.

Root metabolomics data analysis was conducted by Shanghai Majorbio Bio-Pharm Technology Co., Ltd. Raw metabolomic data were subjected to peak extraction, peak alignment, and normalization prior to statistical analysis. Differential metabolites were identified based on partial least squares discriminant analysis (PLS-DA), combined with variable importance in projection (VIP) scores. Model robustness was assessed using cross-validation and 200 permutation tests to minimize the risk of overfitting.

Differential metabolites were screened according to the following criteria: VIP > 1, Student’s t-test (raw *P* < 0.05), and fold change (FC ≥ 1.5 or FC ≤ 0.67). Metabolites with FC ≥ 1.5 were considered upregulated, whereas those with FC ≤ 0.67 were considered downregulated. To reduce the risk of false positives caused by multiple comparisons, false discovery rate (FDR) correction was performed on the raw P values. However, the final identification of differential metabolites was primarily based on the raw P values. PLS-DA was used for supervised classification and variable selection, whereas OPLS-DA was not applied separately to ensure methodological consistency and comparability of results (Fan et al., 2025).

## 3 Results and discussion

### 3.1 Effects of drought stress on growth and root morphology of Hemerocallis minor

#### 3.1.1 Effect on plant height

Drought stress had a significant effect on the plant height of *H. minor* (*P* < 0.05). As shown in **Table 1**, plant height exhibited varying degrees of change under different drought treatments over the observation periods. Compared with the control (CK), mild drought treatment (W1) did not cause significant differences in plant height at weeks 4, 8, and 12 (*P* > 0.05). Moderate (W2), severe (W3), and extreme drought treatments (W4) all affected plant height to varying extents, with W3 and W4 showing significantly lower heights than CK and W1 throughout the observation periods (*P* < 0.05). At weeks 4, 8, and 12, the differences in plant height between W1 and W3 were 55.66%, 53.45%, and 63.30%, respectively, while those between W1 and W4 were 200.27%, 235.00%, and 247.56%, respectively. These results indicate that mild drought did not significantly affect plant height, whereas moderate and more severe drought conditions significantly inhibited growth.

#### 3.1.2 Effect on root morphological parameters

**Figure 2:**
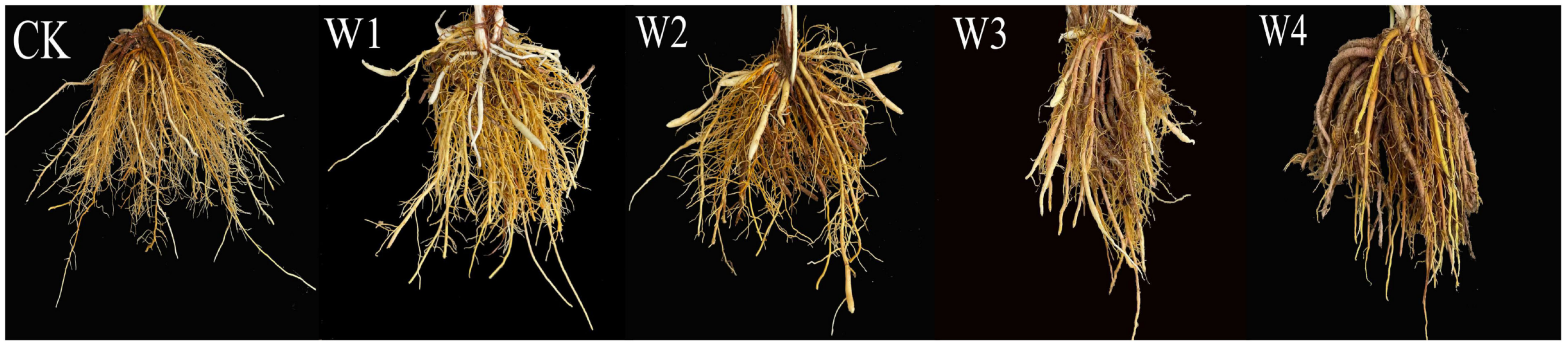
Morphological changes of *Hemerocallis minor* roots under drought stress. All root samples were collected and photographed under the same conditions. The images qualitatively illustrate root morphological changes under different treatments, with thicker roots observed in W1 and W2 treatments. Quantitative analysis of root morphological parameters, including average root diameter, total root length, root volume, and root surface area, is presented in **Table 2**.

Drought stress significantly affected the root morphology of *H. minor*, as shown in **Figure 2**; **Supplementary Table 1** (Comparison of root morphological characteristics of *H. minor* under drought stress). Distinct differences in root external morphology were observed among treatments. Under W1 and W2 treatments, a relatively higher number of thickened roots was observed, and this tendency increased with the severity of drought stress. Meanwhile, the number of newly formed roots remained relatively high in both W1 and W2 treatments, and the overall root system exhibited a comparatively compact structure. Under W3 treatment, the number of new roots decreased, accompanied by a reduction in root branching, indicating a certain degree of restriction in root extension. Under W4 treatment, new root formation was further reduced, the branching pattern became simpler, and overall root growth was markedly inhibited.

These results indicate that drought stress induced significant alterations in root morphology of *H. minor*, characterized by changes in root thickening and branching patterns. However, these morphological variations primarily reflect structural responses and do not directly indicate functional adaptive mechanisms.

#### 3.1.3 Quantitative root parameters

As presented in **Table 2**, drought stress had significant effects on quantitative root parameters of *H. minor* (*P* < 0.05). Root traits differed significantly among treatments and displayed distinct trends with increasing drought intensity. Compared with CK, W1 increased root number, total root length, total root volume, root surface area, and average root diameter by 28.19%, 16.30%, 273.98%, 108.06%, and 78.82%, respectively. In W2, total root volume, root surface area, and average root diameter increased by 111.26%, 36.56%, and 56.16%, respectively. In W3 and W4, all root parameters were generally lower than those in CK, with W4 showing the greatest reduction.

**Table 2:** Effects of drought stress on the morphological parameters of *Hemerocallis minor* roots.

Treatment	Total root number	Total root length (cm)	Total root volume (mm³)	Total root surface area (mm²)	Average root diameter (mm)
CK	26.00 ± 3.00b	76.63 ± 1.94b	26.28 ± 1.23c	477.86 ± 16.21c	0.203 ± 0.003c
W1	33.33 ± 3.51a	89.12 ± 2.95a	98.02 ± 3.08a	994.22 ± 30.87a	0.363 ± 0.003a
W2	19.67 ± 2.08c	68.22 ± 3.55c	55.51 ± 0.70b	652.56 ± 24.57b	0.317 ± 0.007b
W3	13.33 ± 2.31d	24.61 ± 2.56d	4.14 ± 0.31d	104.98 ± 8.32d	0.14 ± 0.010d
W4	9.33 ± 0.58d	15.30 ± 1.57e	1.47 ± 0.24d	49.50 ± 6.69d	0.097 ± 0.003e

Values are presented as mean ± SD (n = 3). Different lowercase letters indicate significant differences among treatments, determined by one-way ANOVA followed by Duncan’s multiple range test (*P* < 0.05).

These results indicate that different levels of drought stress significantly affected the root structure of *H. minor*. Under mild to moderate drought conditions, some root morphological traits increased to a certain extent, whereas severe and extreme drought suppressed overall root growth. These changes primarily reflect morphological responses and do not support direct inferences regarding succulence as a water-storage or functional adaptive mechanism.

### 3.2 Effect of drought stress on physiological traits of Hemerocallis minor leaves and roots

#### 3.2.1 Effect on total chlorophyll content

**Figure 3:**
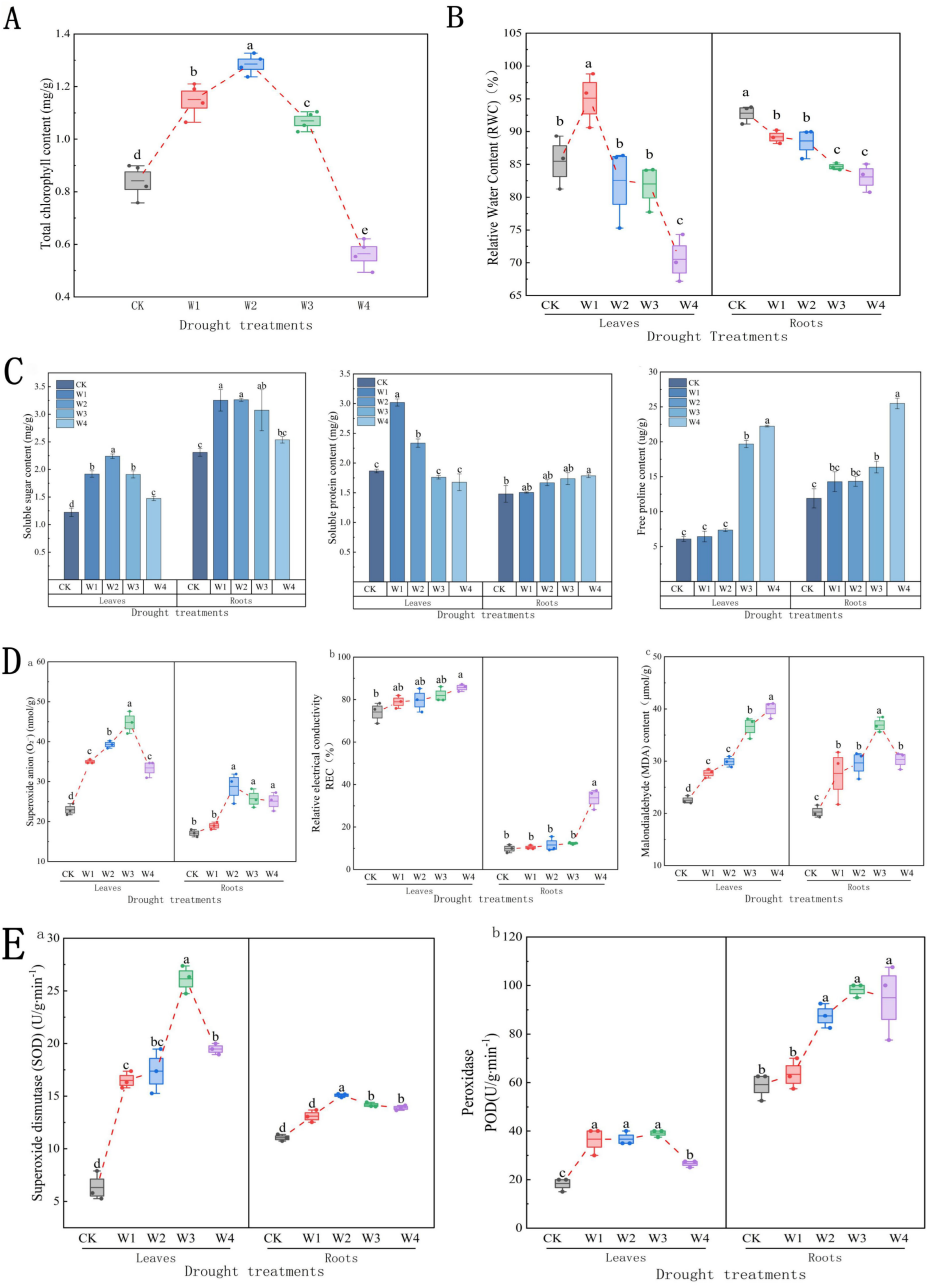
Physiological responses of *Hemerocallis minor* to drought stress. **(A)** Total chlorophyll content; **(B)** Relative water content in leaves and roots; **(C)** Osmoregulatory substances; **(D)** Membrane system-related indicators; **(E)** Antioxidant enzyme activities. Different letters indicate significant differences among treatments (*P* < 0.05). Data are presented as mean ± SD (n=3).

Drought stress significantly affected the total chlorophyll content of *H. minor* leaves (*P* < 0.05). As shown in **Figure 3A**, total chlorophyll content exhibited an overall increase followed by a decrease with increasing drought intensity, reaching the highest value under W2 (1.30 mg·g^-1^) and the lowest under W4 (0.59 mg·g^-1^). Compared with the CK, W2 increased chlorophyll content by 49.56%, while W4 decreased it by 32.45%.

These results indicate that chlorophyll content varied significantly under different drought levels, reflecting the response of the leaf pigment system to water availability. However, chlorophyll content alone cannot be directly used to infer changes in photosynthetic capacity or overall physiological function.

#### 3.2.2 Effect on relative water content

Drought stress significantly influenced the relative water content (RWC) of both leaves and roots of *H. minor* (*P* < 0.05). As shown in **Figure 3B**, leaf RWC exhibited an initial increase followed by a decrease with increasing drought intensity, with the highest value in W1 (95.1%), representing an 11.24% increase relative to CK, and the lowest in W4, which decreased by 17.52% compared with CK. Root RWC showed an overall declining trend, with CK highest (92.8%) and W4 lowest; W1 and W2 decreased by 3.91% and 4.55% relative to CK, respectively.

These results indicate that drought stress differentially affected water status in aboveground and belowground tissues. Changes in leaf and root RWC may reflect dynamic adjustments in water allocation and tissue dehydration, but should not be directly interpreted as evidence of stable physiological adaptation mechanisms.

#### 3.2.3 Effect on osmotic regulator substances

Drought stress significantly affected the contents of osmoregulatory substances in *H. minor* (*P* < 0.05). As shown in **Figure 3C**, soluble protein (SP), soluble sugar (SS), and free proline (Pro) exhibited varying responses in leaves and roots. Leaf SP was higher under W1 and W2, with the highest value in W1 (3.02 mg·g^-1^), representing a 61.82% increase relative to CK. Root SP peaked under W4 (1.78 mg·g^-1^), a 20.67% increase over CK. SS in both leaves and roots reached the highest levels under W2, increasing by 83.11% and 41.27%, respectively, relative to CK. Pro content in both tissues increased markedly with drought intensity, peaking under W4 at 22.24 μg·g^-1^ in leaves and 25.49 μg·g^-1^ in roots.

These results indicate that changes in osmolyte content reflect the physiological responses of *H. minor* to alterations in cellular osmotic potential under different drought conditions. Their increase or decrease may be related to the severity of water stress, but these changes do not necessarily indicate an overall enhancement of stress tolerance and should be interpreted solely as indicators of drought response.

#### 3.2.4 Effect on membrane system-related indicators

Drought stress significantly influenced membrane system-related indicators of *H. minor* (*P* < 0.05). As shown in **Figure 3D**, superoxide anion (O_2_-), relative electrical conductivity (REC), and malondialdehyde (MDA) exhibited significant changes among treatments. Leaf O_2_- content peaked under W3 (44.82 nmol·g^-1^), a 95.3% increase over CK, while root O_2_- peaked under W2 (28.8 nmol·g^-1^), 68.47% higher than CK. REC increased in both leaves and roots with drought intensity, reaching maxima under W4 (85.66% and 33.73%), representing increases of 15.56% and 243.13% relative to CK, respectively. MDA content in leaves was highest under W4 (40.03 μmol·g^-1^), a 77.82% increase over CK; root MDA peaked under W3 (30.32 μmol·g^-1^), an 82.3% increase.

These results indicate that drought stress induces reactive oxygen species accumulation and enhances lipid peroxidation. The increases in O_2_-, REC, and MDA primarily reflect the degree of cellular oxidative stress and membrane destabilization, rather than an enhancement of drought tolerance. These damaging effects were more pronounced under severe and extreme drought conditions.

#### 3.2.5 Effect on antioxidant enzyme activity

Drought stress significantly affected antioxidant enzyme activities of *H. minor* (*P* < 0.05). As shown in **Figure 3E**, superoxide dismutase (SOD) and peroxidase (POD) activities varied markedly in different tissues. Leaf SOD activity peaked under W3 (26.14 U·g^-1^·min^-1^), representing a 313.89% increase over CK; root SOD activity peaked under W2 (15.09 U·g^-1^·min^-1^), a 36.6% increase, and decreased slightly under more severe stress, though W3 and W4 remained higher than CK. Leaf POD activity peaked under W3 (39.17 U·g^-1^·min^-1^), a 113.64% increase, but decreased by 31.91% in W4. Root POD activity increased with drought intensity, reaching the maximum under W4 (95 U·g^-1^·min^-1^), a 60.56% increase over CK.

These results indicate that changes in antioxidant enzyme activities reflect regulatory responses to reactive oxygen species accumulation under drought stress. The increase or decrease in enzyme activities may be associated with stress intensity and the degree of tissue damage. However, without supporting evidence from gene expression or protein-level analyses, these results should not be directly interpreted as an enhancement of drought tolerance, but rather as indicators of oxidative stress response processes.

### 3.3 Effects of drought stress on root metabolites of Hemerocallis minor

#### 3.3.1 Principal component analysis of metabolites

**Figure 4:**
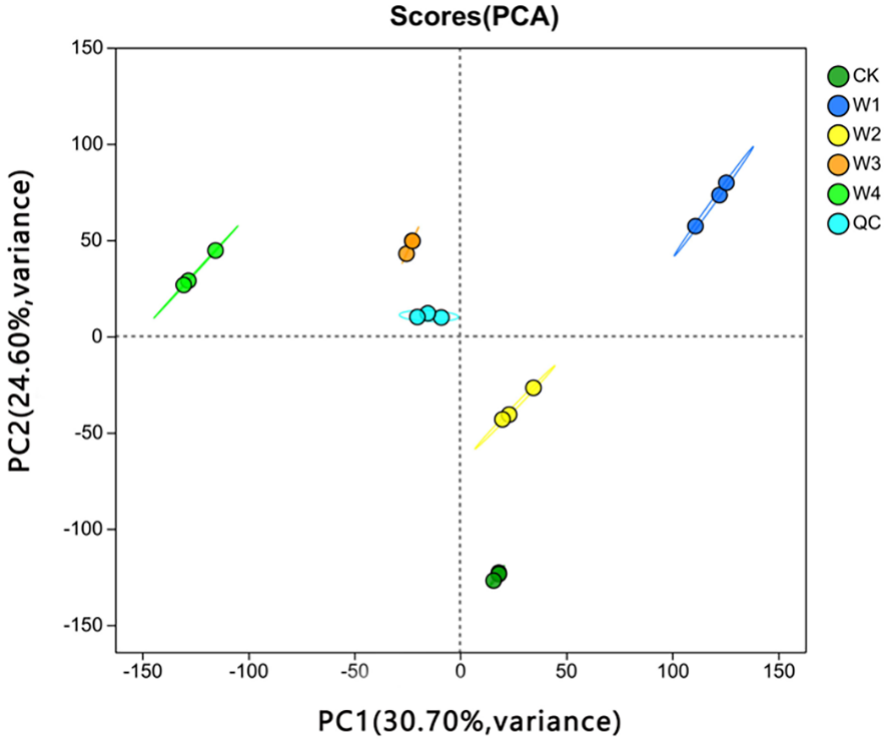
Analysis of root metabolic components (PCA) of *Hemerocallis minor* under different drought stress treatments. Each point represents a biological replicate (n=3). Only statistically supported 95% confidence ellipses are shown.

As shown in **Figure 4**, principal component analysis (PCA) indicated good clustering of quality control (QC) samples, suggesting that the detected data were stable and reliable. PC1 and PC2 explained 30.7% and 24.6% of the total variance, respectively, with a cumulative contribution of 55.3%. Samples under different drought treatments showed a certain degree of separation in the score plot, indicating variations in metabolite profiles among treatments, while the intra-group replicates were closely clustered, reflecting good experimental reproducibility.

In addition, the PCA was performed based on all detected metabolic features for dimensionality reduction, aiming to reflect overall variations in the metabolomic profiles rather than a subset of annotated or pre-selected metabolites.

#### 3.3.2 Metabolite annotation and functional classification

To systematically characterize the metabolite composition, annotation analyses were conducted based on the Kyoto Encyclopedia of Genes and Genomes (KEGG) and the Human Metabolome Database (HMDB). Different statistical outputs corresponded to distinct analytical levels, including raw detected features, database-annotated metabolites, and pathway-mapped results, which were clearly distinguished from one another.

**Figure 5:**
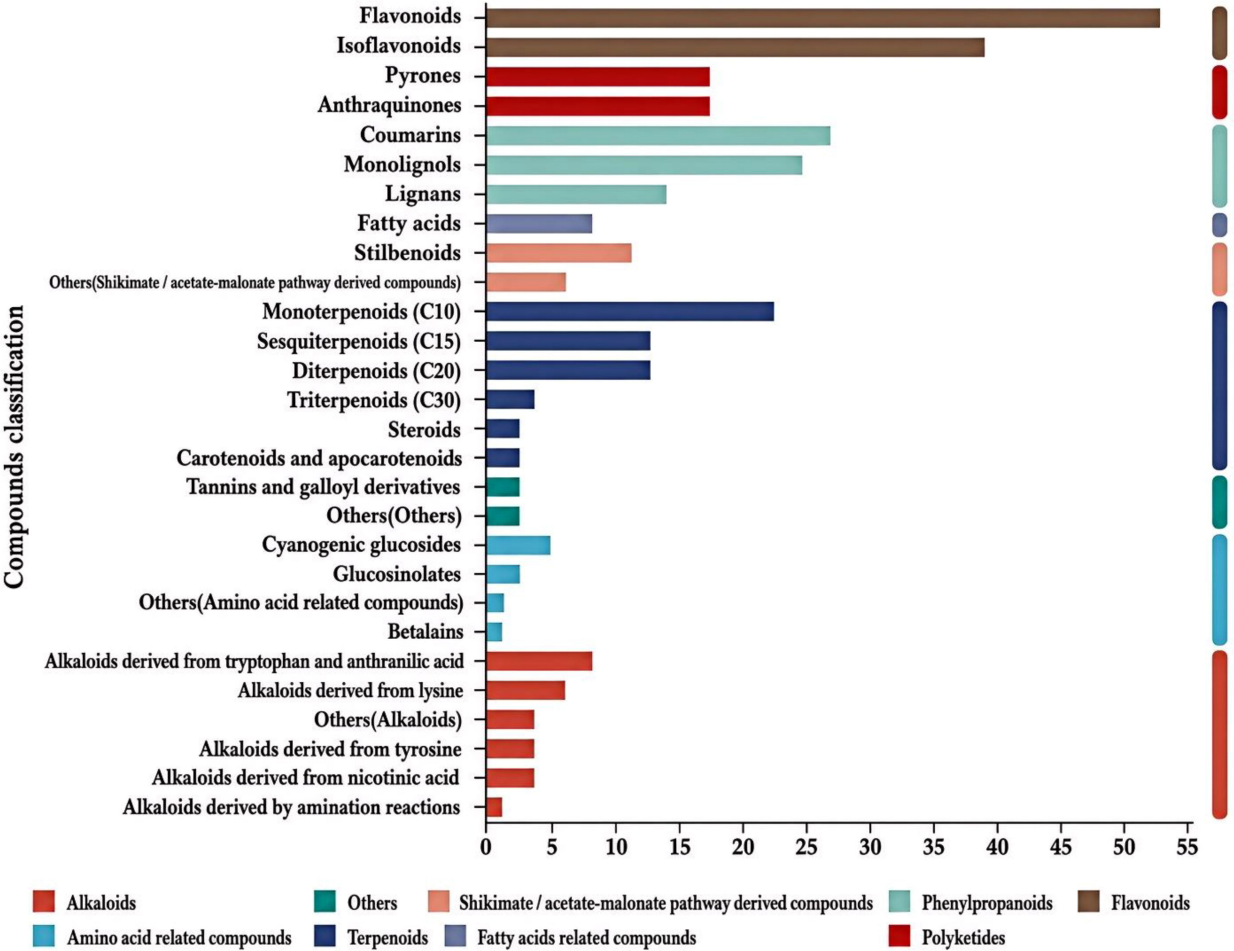
KEGG compound classification statistics. The *y*-axis represents KEGG compound categories, and the *x*-axis represents the number of compounds annotated to each category. Bar colors indicate the corresponding primary (Level 1) compound classifications.

As shown in **Figure 5**, a total of 276 metabolites were annotated in the KEGG Compound database. This KEGG annotation subset, based on chemical structure matching, reflects the classification of metabolites within the KEGG compound system and does not represent all detected metabolic features. At the first classification level, metabolites were assigned to nine major categories, among which flavonoids, phenylpropanoids, and polyketides were the most abundant, with 83, 58, and 30 metabolites, respectively. At the second classification level, 28 metabolite classes were annotated, with flavonoids, isoflavonoids, coumarins, monolignols, and monoterpenoids being the most represented (48, 35, 24, 22, and 20 metabolites, respectively).

**Figure 6:**
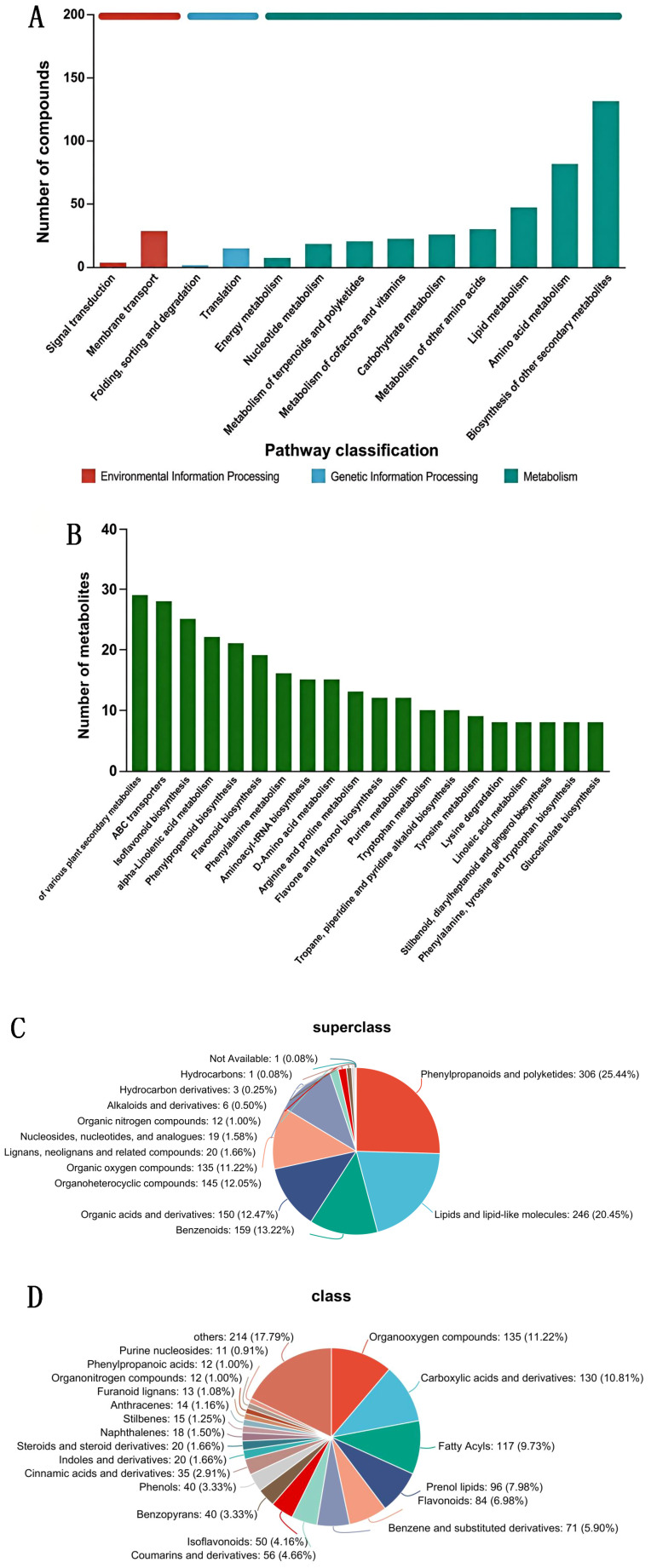
KEGG and HMDB annotation of differential metabolites. **(A)** KEGG pathway statistics; **(B)** Important metabolic pathway statistics; **(C)** HMDB secondary classification; **(D)** HMDB tertiary classification.

As shown in **Figure 6A**, KEGG pathway analysis annotated 430 metabolites into three major functional categories: Metabolism (383 metabolites), Environmental Information Processing (31 metabolites), and Genetic Information Processing (16 metabolites). These 430 metabolites represent the subset successfully mapped to the KEGG Pathway database and correspond to the pathway-level functional annotation, reflecting the biological roles of the metabolites. **Figure 6B** shows that the top 20 enriched pathways were primarily associated with the biosynthesis of various plant secondary metabolites, ABC transporters, and isoflavonoid biosynthesis, among other key metabolic processes.

As illustrated in **Figures 6C, D**, HMDB annotation assigned 1,203 metabolites to chemical classes. This HMDB-based structural classification describes the distribution of metabolites across chemical categories and represents a different database layer from KEGG annotation and pathway mapping. Among these, the eight most abundant classes were: organooxygen compounds (135 metabolites, 11.22%), carboxylic acids and derivatives (130, 10.81%), fatty acyls (117, 9.73%), prenol lipids (96, 7.98%), flavonoids (84, 6.98%), benzene and substituted derivatives (71, 5.9%), coumarins and derivatives (56, 4.66%), and isoflavonoids (50, 4.16%). The high proportion of organooxygen compounds, carboxylic acids and derivatives, and fatty acyls suggests that primary metabolism in *H. minor* roots is relatively active, mainly involving energy metabolism, lipid metabolism, and carbon-nitrogen metabolic processes.

#### 3.3.3 Screening and identification of differential metabolites

Differential metabolites were screened using the variable importance in projection (VIP) scores from the PLS-DA model, combined with statistical significance (raw *P* < 0.05) and fold change (FC ≥ 1.5 or FC ≤ 0.67). A VIP > 1 indicates a metabolite’s important contribution to group discrimination, *P* < 0.05 indicates statistically significant changes, and FC ≥ 1.5 or FC ≤ 0.67 represents relative up- or downregulation, respectively.

As shown in **Table 3**, a total of 1,349 significant differential metabolites were detected. This set represents statistically screened differential features reflecting the intensity of metabolic responses between treatments, but does not correspond to the total number of KEGG- or HMDB-annotated metabolites. Overall, across different analytical layers, this study obtained 1,349 differential metabolic features, 276 KEGG compound annotations, 430 KEGG pathway-mapped metabolites, and 1,203 HMDB-classified metabolites, corresponding to four hierarchical levels: “differential screening-compound annotation-pathway mapping-structural classification.”.

**Table 3:** Differential metabolite numbers of roots under different drought stress treatments.

Group	Total identified compounds	Total significantly differential metabolites	Significantly upregulated metabolites	Significantly downregulated metabolites
G1(W1vsCK)	6177	553	177	376
G2(W2vsCK)	6080	533	230	303
G3(W3vsCK)	6155	503	290	213
G4(W4vsCK)	6536	595	325	270
G5(W2vsW1)	6039	600	369	231
G6(W3vsW1)	6081	609	469	140
G7(W4vsW1)	6279	566	378	188
G8(W3vsW2)	5851	620	413	207
G9(W4vsW2)	6306	644	379	265
G10(W4vsW3)	6008	589	279	310

The number of differential metabolites per sample group ranged from 503 to 644, with 177–469 significantly upregulated and 140–376 downregulated metabolites. Specifically, the G1 group contained 553 differential metabolites, including 177 upregulated and 376 downregulated; G2 contained 533 metabolites, including 230 upregulated and 303 downregulated; G3 contained 503 metabolites, including 290 upregulated and 213 downregulated. The distribution of differential metabolites in groups G4-G10 is also provided in **Table 3**.

**Figure 7:**
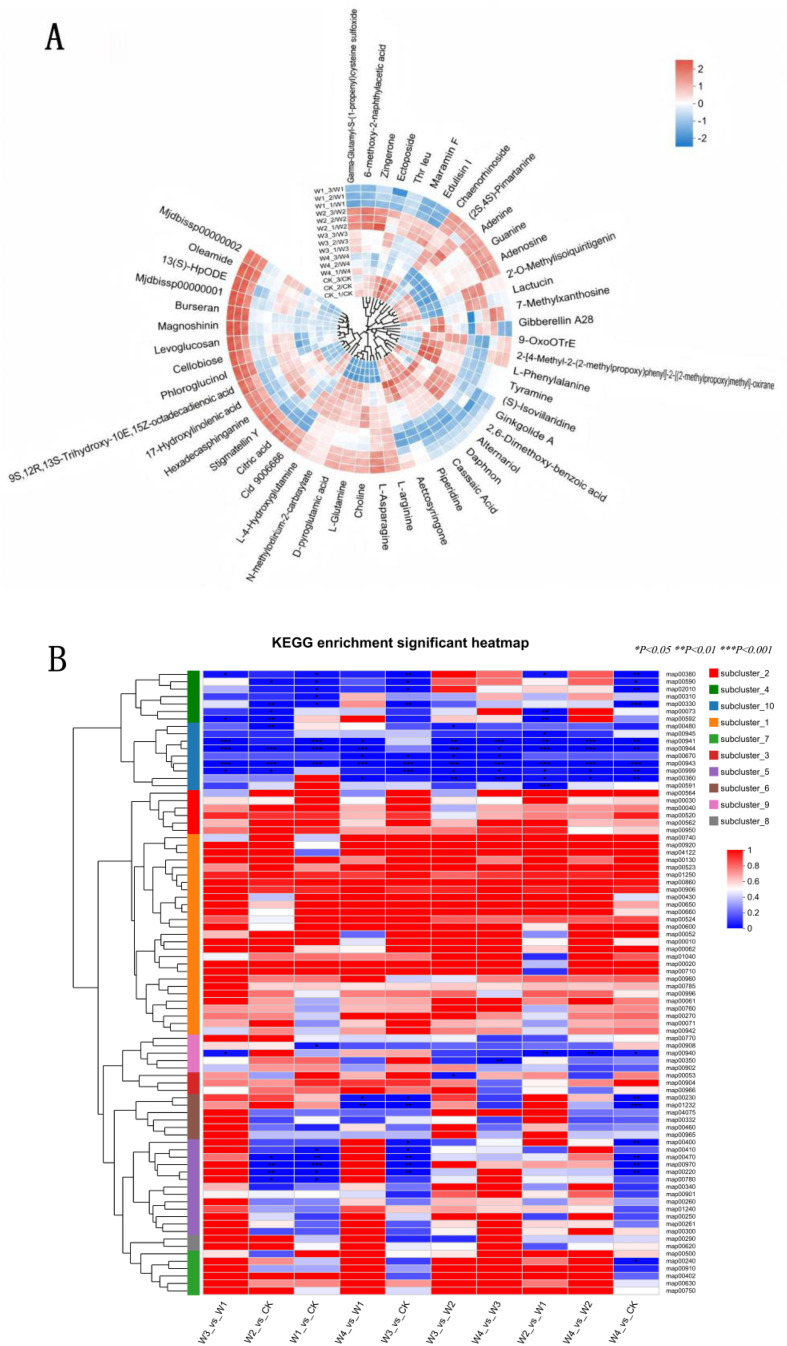
Clustering and KEGG pathway enrichment analyses of differential metabolites in Hemerocallis minor under drought stress. **(A)** Clustering heat map of differential metabolites. **(B)** KEGG pathway enrichment analysis of differential metabolites. The top enriched pathways are shown with corresponding enrichment significance (*P* < 0.05).

As shown in **Figure 7A**, hierarchical clustering analysis (HCA) reveals the expression patterns of differential metabolites across different sample groups. The heatmap colors represent relative metabolite abundance, while the dendrogram indicates clustering relationships among metabolites. At the plant primary classification level, 12 major categories of differential metabolites were identified, including amino acids and derivatives (e.g., L-phenylalanine), coumarins and derivatives (e.g., edulisin I), vitamins (e.g., adenine), flavonoids (e.g., 2′-O-methylisoliquiritigenin), terpenoids (e.g., gibberellin A28), and quinones (e.g., danthron). Overall, drought stress significantly altered the root metabolome of *H. minor*, with enrichment in amino acids, flavonoids, terpenoids, and organic acids.

#### 3.3.4 KEGG pathway enrichment analysis of differential metabolites

To further investigate the biological functions of differential metabolites, KEGG pathway enrichment analysis was performed on the metabolites screened in this study to explore their potential roles in metabolic and signaling pathways. Differential metabolites were first selected based on VIP > 1, *P* < 0.05, and fold change (FC ≥ 1.5 or ≤ 0.67), and then mapped to KEGG pathways for enrichment analysis. As shown in **Figure 7B**; **Supplementary Table 2** (Significant enrichment pathways of differential metabolites), a total of 29 significantly enriched pathways were identified, including arginine and proline metabolism, flavonoid biosynthesis, phenylalanine metabolism, aminoacyl-tRNA biosynthesis, arachidonic acid metabolism, and flavone and flavonol biosynthesis. Among these, the isoflavonoid biosynthesis pathway was the most significantly enriched (*P* < 0.001), with 23 differential metabolites mapped to this pathway. These results represent a KEGG Pathway-level mapping subset and reflect the functional distribution of differential metabolites within the metabolic network.

#### 3.3.5 Isoflavonoid-related metabolites

**Figure 8:**
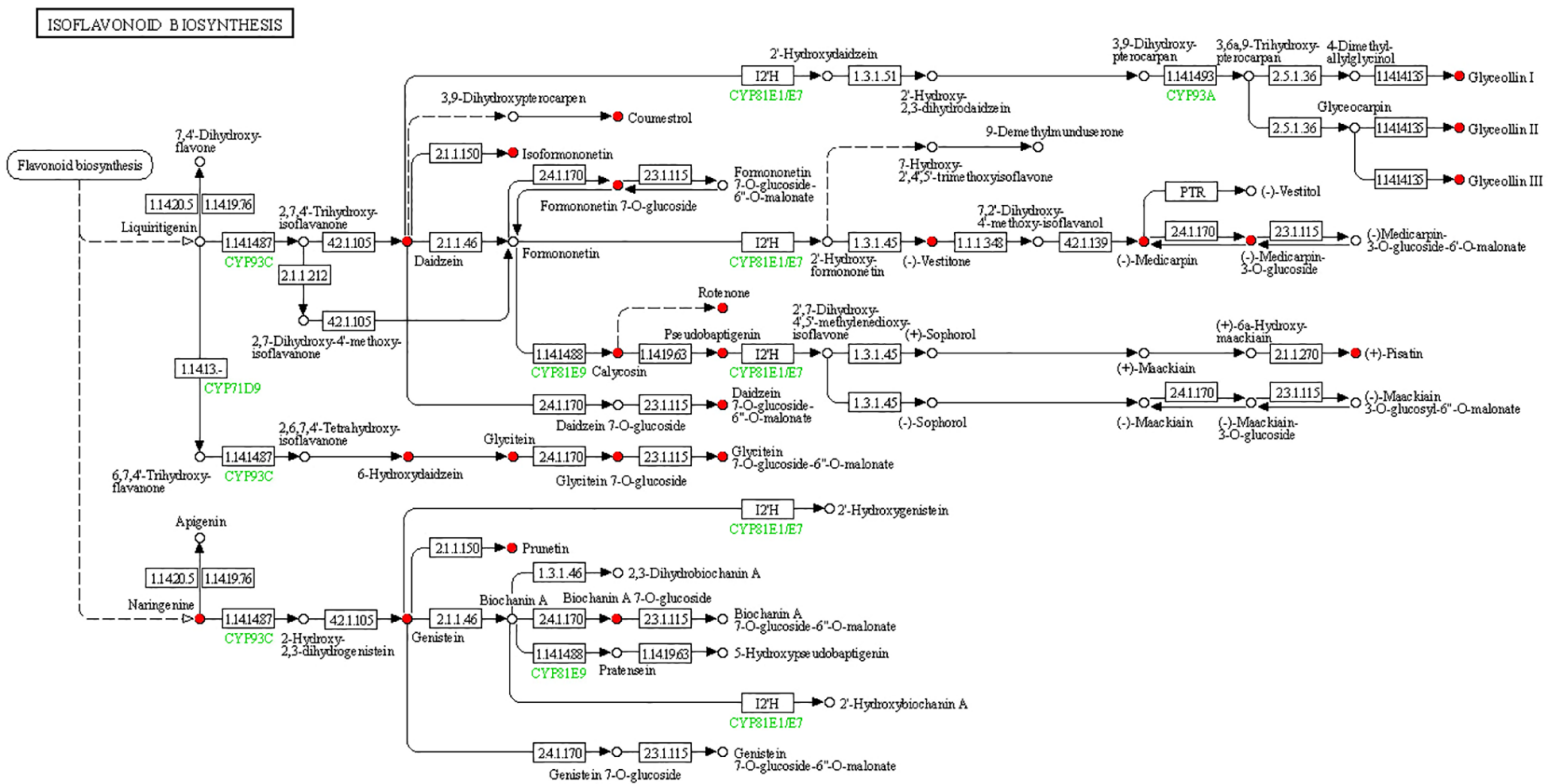
Isoflavone biosynthesis pathway diagram. Red solid circles indicate up-regulated metabolites, and hollow circles indicate down-regulated metabolites. Each metabolite is annotated according to the KEGG pathway database. Arrows represent enzymatic reactions, with enzyme codes (EC numbers) indicated in rectangles. Node size corresponds to relative abundance, and values represent fold change (mean ± SD, n=3).

Based on KEGG compound ID–based pathway enrichment analysis, 23 significantly differential metabolites were identified in the isoflavonoid biosynthesis pathway (**Figure 8**), including (−)-medicarpin, coumestrol, vestitone, rotenone, glyceollin III, sissotrin, daidzein, genistein, and 6″-O-malonyldaidzin. As shown in **Supplementary Table 3** (Differential metabolite regulation annotated to the isoflavone biosynthesis pathway), metabolites such as pisatin, naringenin, genistein, and 6″-O-malonyldaidzin exhibited an overall upregulation trend under drought stress, whereas (−)-medicarpin, vestitone, and glyceollin III showed a decreasing trend within the isoflavonoid biosynthesis pathway. It should be noted that *H. minor* is not a typical legume species; the identified isoflavonoid metabolites require further confirmation through high-confidence MS/MS matching, standards verification, or targeted LC-MS/MS. In the absence of such validation, this study reports only the identification and abundance changes of metabolites, without treating the isoflavonoid biosynthesis pathway as a confirmed regulatory mechanism.

### 3.4 Correlation analysis between plant morphological parameters, physiological traits, and differential metabolites

#### 3.4.1 Correlation analysis between morphological parameters and physiological indicators

**Figure 9:**
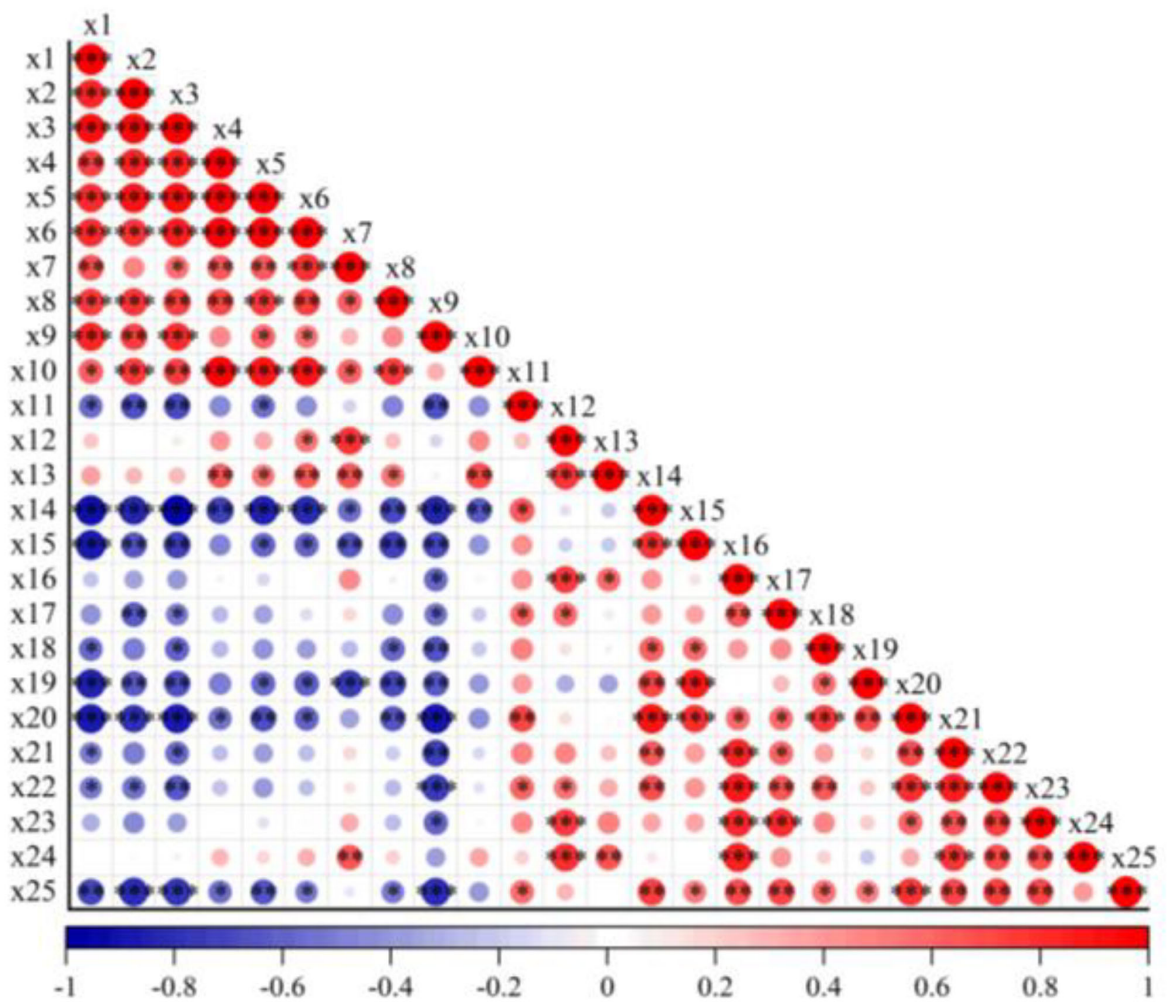
Correlation analysis between growth morphology and physiological indexes of *Hemerocallis minor* under drought stress. *P <= 0.05, **P <= 0.01, ***P <= 0.001;x1, plant height; x2, root number; x3, total root length; x4, total root volume; x5, total root surface area; x6, average root diameter; x7, chlorophyll content; x8, leaf RWC; x9, root RWC; x10, leaf SP; x11, root SP; x12, leaf SS; x13, root SS; x14, leaf Pro; x15, root Pro; x16, leaf O_2_^-^; x17, root O_2_^-^; x18, leaf relative conductivity; x19, root relative conductivity; x20, leaf MDA; x21, root MDA; x22, leaf SOD; x23, root SOD; x24, leaf POD; x25, root POD. The same applies below.

To investigate the relationships between morphological parameters and physiological traits of *H. minor*, Pearson correlation analysis was performed on 25 traits using biological replicates from all treatments, with p-values adjusted for multiple testing (**Figure 9**).

The results indicated that plant height, root number, total root length, total root volume, total root surface area, and average root diameter were significantly or highly positively correlated, suggesting a consistent variation trend among different root morphological parameters. Plant height and root-related traits were positively correlated with leaf RWC and chlorophyll content, whereas negative correlations were observed with certain stress-responsive indicators, including Pro, MDA, and REC, indicating a relationship between plant growth status, water status, and stress response. Furthermore, O_2_-, SOD, and POD in leaves and roots generally exhibited positive correlations, while MDA showed partial correlations with antioxidant enzyme activities, suggesting coordinated variation among oxidative stress-related indicators.

Overall, complex correlations exist among morphological, physiological, and antioxidant traits; however, correlation analysis reflects only statistical associations and cannot be interpreted as direct causal relationships.

#### 3.4.2 Correlation analysis between morphological–physiological indicators and differential metabolites

**Figure 10:**
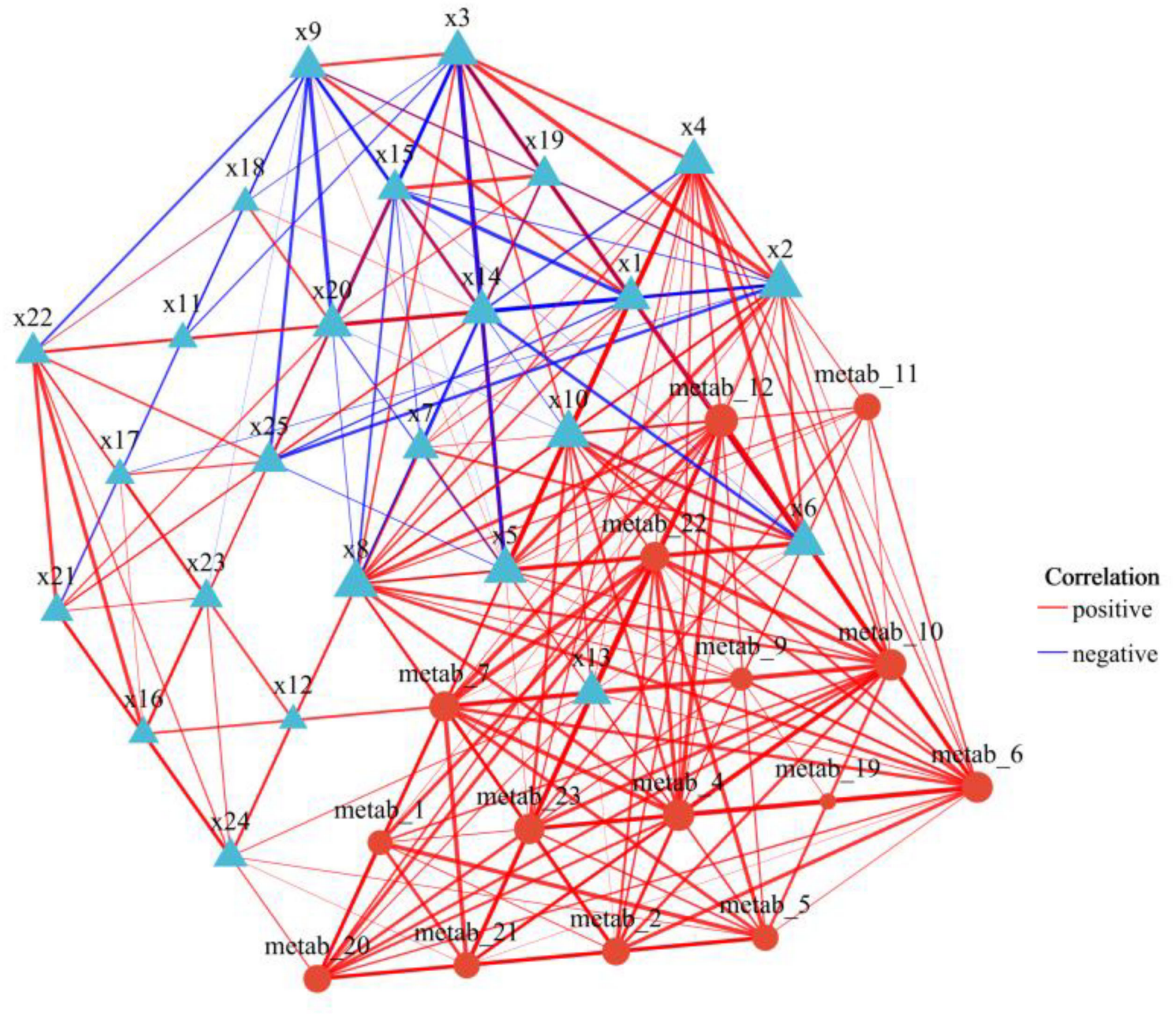
Correlation analysis between morphological and physiological indexes and differential metabolites of *Hemerocallis minor* under drought stress. Edges represent significant correlations with |r| > 0.7, *P* < 0.05, adjusted by the Benjamini-Hochberg method. Node size reflects metabolite degree, and edge thickness indicates correlation strength.

Pearson correlation analysis was conducted to investigate the relationships between selected differential metabolites and morphophysiological traits (**Figure 10**). Correlation networks were constructed using a threshold of |r| > 0.7 and Benjamini-Hochberg adjusted *P* < 0.05. The analysis was based on raw data from biological replicates of each treatment (rather than treatment means) to avoid reduced degrees of freedom and inflated correlation estimates caused by averaging. The metabolites shown in **Figure 10** represent a subset of differential metabolites, selected for correlation analysis based on VIP > 1, *P* < 0.05, and fold change (FC ≥ 1.5 or ≤ 0.67), and correspond to those annotated in KEGG pathways.

The analysis revealed significant correlations between certain isoflavonoids and related metabolites with leaf RWC, root number, total root volume, root surface area, SP, and SS. For example, coumestrol, sissotrin, and 6’’-O-malonyl-daidzin were significantly positively correlated with leaf RWC, whereas some flavonoid metabolites were positively correlated with root morphological parameters. In addition, specific metabolites showed correlations with POD and REC.

These results suggest that changes in differential metabolites may be associated with plant water status, root morphology, and antioxidant responses. Nevertheless, as correlation analysis cannot directly reveal the biological functions of metabolites, the current findings should be interpreted as exploratory evidence of potential metabolite-trait associations. Further targeted metabolomic validation and functional experiments are required to elucidate the underlying regulatory mechanisms.

### 3.5 Discussion

This study demonstrated that *H. minor* maintains a relatively stable aboveground growth under mild drought, with slight increases in certain root traits, whereas severe and extreme drought significantly inhibited plant height, growth rate, and root morphology, exhibiting a stepwise response pattern of “moderate drought promotion, excessive drought inhibition,” consistent with findings in desert herbaceous plants (Jiang et al., 2017). Under mild drought, *H. minor* did not exhibit apparent compensatory growth, suggesting a strategy focused on maintaining stability rather than rapid biomass accumulation. Regarding root morphology, mild drought significantly increased root number, total root length, and average root diameter, while these parameters gradually declined with increasing drought severity, and were markedly lower than the control under extreme drought, indicating a potential threshold in root morphological plasticity. The formation of fleshy roots under drought may buffer water deficit by increasing root diameter and water-storage tissue proportion; however, their functional potential appears limited under severe and extreme drought. Root anatomical validation was not conducted in this study, and increased root diameter may also reflect developmental thickening or restricted elongation rather than adaptive succulence per se. These observations align with reports on sorghum and soybean root responses (Ding et al., 2013; Jeevitha et al., 2022), suggesting that *H. minor* exhibits plasticity in root morphology under drought, which could inform ecological restoration applications, although specific adaptive roles require experimental confirmation.

At the physiological level, *H. minor* exhibited stage-dependent responses under different drought intensities. Chlorophyll content showed a hump-shaped response, increasing under moderate drought and decreasing under extreme drought (Ren et al., 2016). Leaf and root RWC were higher under mild drought but decreased significantly with increasing drought severity, with the greatest reduction under extreme drought (Sun et al., 2023). Notably, leaf RWC was slightly higher than the control under mild drought, concomitant with an increase in leaf SP, reflecting short-term physiological adjustments such as optimized water allocation and enhanced osmolyte accumulation to maintain cellular water homeostasis rather than an overall increase in drought tolerance. Osmoregulators including SP, SS, and Pro accumulated under drought, yet elevated Pro, MDA, REC, and antioxidant enzyme activities may also indicate stress-induced damage rather than necessarily improved drought resistance (Hill et al., 2020). Increases in reactive oxygen species (O_2_-) and MDA, together with elevated REC, indicate oxidative damage to cell membranes, which was particularly pronounced under severe and extreme drought (Chang et al., 2018). Leaf O_2_- increased under mild to moderate drought but decreased under severe and extreme drought, possibly due to inhibited photosynthesis, reduced electron transport, and enhanced antioxidant enzyme activity. SOD and POD activities exhibited a gradient response, suggesting attempts to regulate antioxidant capacity and mitigate ROS accumulation; however, gene expression or enzyme abundance were not assessed, and thus these physiological responses represent observational phenomena rather than confirmed regulatory mechanisms (Araz et al., 2025). Overall, *H. minor* exhibited a stage-dependent response transitioning from “adaptive adjustment” to “stress tolerance” and ultimately to “physiological limitation,” with specific drought mechanisms requiring further verification.

At the metabolic level, drought stress substantially altered the root metabolome of *H. minor*, primarily affecting amino acids and derivatives, flavonoids, coumarins, organic acids, lipids, and phenolic acids, consistent with drought studies in other plants (Liu, 2024). Pathway enrichment analysis indicated significant enrichment in the isoflavonoid biosynthesis pathway, consistent with reports in soybean and maize (Wang et al., 2022, Wang et al., 2023). Isoflavonoids may participate in ROS scavenging, hormone regulation, and defense responses, yet targeted validation or molecular pathway analysis was not performed, so their roles remain speculative. Accumulation of isoflavonoids may also reflect stress responses or metabolic reallocation rather than a definitive drought adaptation mechanism.

Integrating morphological, physiological, and metabolic results, a working hypothesis of “root structural plasticity-physiological homeostasis regulation- enhanced metabolic defense” is proposed to explain the multilayered response of *H. minor* to drought stress (Franco-Navarro et al., 2025). Under mild drought, root morphological adjustments and physiological adaptation may dominate the response, whereas under severe drought, metabolite accumulation, including isoflavonoids, may contribute to mitigating oxidative stress or maintaining root function. This hypothesis requires further verification through root anatomical analysis, multi-omics integration, and hormone or enzyme activity assays (Wang et al., 2024c; Qian et al., 2025). As a drought-tolerant industrial ecological restoration plant, *H. minor* exhibits strong stress resistance and stable metabolic traits and has been applied in food, medicinal, and landscape ecological contexts. This study provides a theoretical basis for its large-scale cultivation and ecological utilization in drought-prone regions, although the underlying molecular mechanisms warrant further investigation.

## 4 Conclusions

The results of this study demonstrate that drought stress exerts significant effects on the morphology, physiology, and metabolism of *H. minor*. Morphologically, mild drought maintained plant growth, with certain root traits, such as root diameter and total root length, showing moderate changes, whereas severe and extreme drought markedly inhibited plant height and root development. Physiologically, plants partially maintained internal homeostasis under mild stress, but severe drought intensified membrane damage and oxidative stress, indicating a shift from growth maintenance to defensive adjustments. Metabolically, drought substantially reprogrammed the root metabolic network, with differential metabolites predominantly enriched in stress-related pathways, including amino acid metabolism, flavonoid/isoflavonoid biosynthesis, and secondary metabolite pathways. Although isoflavonoid biosynthesis exhibited notable changes, metabolite identification requires targeted validation, particularly in non-leguminous species. Certain key metabolites were correlated with root morphological and physiological traits, but their specific functions and regulatory roles remain to be verified, as evidence from transcriptomics, hormone analyses, root anatomy, photosynthesis, or functional recovery is currently lacking.

Overall, mild drought preserved partial growth and root traits, whereas severe drought induced more pronounced physiological and metabolic stress responses. These findings provide preliminary insights for the potential application of drought-tolerant plants in industrial ecological restoration. However, given the limitations of greenhouse conditions, sample size, and trait measurements, future studies should integrate field validation, succulent root anatomical analysis, targeted LC-MS/MS quantification of key metabolites, and rehydration recovery experiments (including biomass restoration, survival rate, and root water storage capacity) to comprehensively elucidate the drought-adaptive characteristics of *H. minor*.

## Data Availability

The original contributions presented in the study are included in the article/**Supplementary Material**. Further inquiries can be directed to the corresponding authors.
