# Supplementary material for: Morphological, physiological, and root metabolomic responses of Hemerocallis minor Mill. to drought stress
Source: Front Plant Sci. 2026 Jul 16;17:1855949. doi: 10.3389/fpls.2026.1855949 (PMC13422142; doi:10.3389/fpls.2026.1855949)
Supplement: Supplementary file 1 [file Supplementaryfile1.docx]

Table S1 Comparison of root morphological characteristics of *Hemerocallis minor* under drought stress

| Treatment | Succulent roots | Root color | Adventitious root number | | Root vitality |
| --- | --- | --- | --- | --- | --- |
| CK | Absent | Light yellow-brown | | High | Healthy |
| W1 | Slight formation | Pale yellow | | High | Vigorous |
| W2 | Markedly increased | Yellow-brown | | Moderate | Active |
| W3 | Reduced | Dark yellow-brown | | Low | Inhibited |
| W4 | Rare/absent | Dark brown | | Very low | Severely inhibited |

**Note:** The degree of fleshy root formation was evaluated using a visual semi-quantitative grading method based on external morphological indicators, including root thickening, tissue swelling characteristics, and root color changes.

Table S2 Significant enrichment pathways of differential metabolites

| Pathway ID | Pathway_Description |
| --- | --- |
| map00592 | alpha-Linolenic acid metabolism |
| map00380 | Tryptophan metabolism |
| map00310 | Lysine degradation |
| map00073 | Cutin, suberine and wax biosynthesis |
| map00999 | Biosynthesis of various plant secondary metabolites |
| Pathway ID | Pathway_Description |
| map00590 | Arachidonic acid metabolism |
| map02010 | ABC transporters |
| map00941 | Flavonoid biosynthesis |
| map00944 | Flavone and flavonol biosynthesis |
| map00360 | Phenylalanine metabolism |
| map00591 | Linoleic acid metabolism |
| map00970 | Aminoacyl-tRNA biosynthesis |
| map00053 | Ascorbate and aldarate metabolism |
| map00230 | Purine metabolism |
| map00350 | Tyrosine metabolism |
| map00400 | Phenylalanine, tyrosine and tryptophan biosynthesis |
| map00670 | One carbon pool by folate |
| map00940 | Phenylpropanoid biosynthesis |
| map00480 | Glutathione metabolism |
| map00945 | Stilbenoid, diarylheptanoid and gingerol biosynthesis |
| map00470 | D-Amino acid metabolism |
| map00780 | Biotin metabolism |
| map00908 | Zeatin biosynthesis |
| map00220 | Arginine biosynthesis |
| map00330 | Arginine and proline metabolism |
| map00240 | Pyrimidine metabolism |
| map00943 | Isoflavonoid biosynthesis |
| map01232 | Nucleotide metabolism |
| map00410 | beta-Alanine metabolism |

Table S3 Differential metabolite regulation annotated to the isoflavone biosynthesis pathway

| Group | Down | Up |
| --- | --- | --- |
| G1（W1vsCK） | (-)-medicarpin、Coumestrol、Vestitone、6,7,4'-trihydroxyisoflavone、Rotenone、Glyceollin III、Sissotrin、Daidzein、Genistein、6''-O-Malonyldaidzin、Glycitin、Pseudobaptigenin、Glycitein | Pisatin |
| G2（W2vsCK） | Glyceollin II、6''-O-Malonyldaidzin、Pseudobaptigenin、Medicocarpin、(-)-Glyceollin I | Coumestrol、Prunetin、6,7,4'-trihydroxyisoflavone、Calycosin、Sissotrin、Genistein、Glycitin、Glycitein |
| G3（W3vsCK） | (-)-medicarpin、Vestitone、Glyceollin III | 6''-O-Malonyldaidzin、Pseudobaptigenin、Naringenin、Isoformononetin、Pisatin |
| G4（W4vsCK） | (-)-medicarpin、Vestitone、Glyceollin III、Sissotrin、6''-O-Malonylglycitin、Glycitin、Glycitein | Prunetin、6''-O-Malonyldaidzin、Pseudobaptigenin、Naringenin、Isoformononetin、Pisatin |
| G5（W2vsW1） | Glyceollin II、(-)-Glyceollin I、Isoformononetin | (-)-medicarpin、Coumestrol、Vestitone、Prunetin、6,7,4'-trihydroxyisoflavone、Rotenone、Glyceollin III、Calycosin、Sissotrin、Daidzein、Genistein、Glycitin、Glycitein |
| G6（W3vsW1） | / | Coumestrol、Prunetin、6,7,4'-trihydroxyisoflavone、Ononin、Sissotrin、Genistein、6''-O-Malonyldaidzin、Glycitin、Pseudobaptigenin、Naringenin、Glycitein、Isoformononetin、Pisatin |
| G7（W4vsW1） | 6''-O-Malonylglycitin | Coumestrol、Prunetin、6,7,4'-trihydroxyisoflavone、Rotenone、Daidzein、Genistein、6''-O-Malonyldaidzin、Pseudobaptigenin、Medicocarpin、Isoformononetin、Pisatin |
| G8（W3vsW2） | (-)-medicarpin、Coumestrol、Vestitone、Prunetin、6,7,4'-trihydroxyisoflavone、Glyceollin III、Calycosin、Genistein、Glycitin、Glycitein | Ononin）、Glyceollin II、6''-O-Malonyldaidzin、Pseudobaptigenin、Naringenin、Medicocarpin、（(-)-Glyceollin I、Isoformononetin、Pisatin |
| G9（W4vsW2） | (-)-medicarpin、6,7,4'-trihydroxyisoflavone、Glyceollin III、Calycosin、Sissotrin、Genistein、6''-O-Malonylglycitin、Glycitin、Glycitein | Prunetin、Glyceollin II、6''-O-Malonyldaidzin、Pseudobaptigenin、Naringenin、Medicocarpin、Isoformononetin、Pisatin |
| G10（W4vsW3） | 6,7,4'-trihydroxyisoflavone、Ononin、Sissotrin、Genistein、6''-O-Malonylglycitin、Glycitin、Glycitein | Prunetin、Rotenone、Daidzein、6''-O-Malonyldaidzin、Pseudobaptigenin |
